# Pulmonary epithelioid haemangioendothelioma: A comprehensive review of clinical and molecular advances

**DOI:** 10.1016/j.clinsp.2025.100789

**Published:** 2025-10-13

**Authors:** Bo Tang, Shengliang Zhao, Jingsi Wang, Mingzhang Xiang, Jigang Dai, Quanxing Liu

**Affiliations:** Army Medical University Xinqiao Hospital, Chongqing, China

**Keywords:** Pulmonary epithelioid haemangioendothelioma, Clinical features, Diagnosis, Treatment, Prognosis

## Abstract

**Background:**

Pulmonary Epithelioid Haemangioendothelioma (PEH) is a rare vascular tumor with specific clinical symptoms (e.g., cough, chest pain) and imaging manifestations (multiple nodules in both lungs) that can be easily misdiagnosed or lead to a poor prognosis.

**Objective:**

To systematically analyse the pathological features, diagnostic challenges, and therapeutic strategies of PEH to provide evidence for its clinical management.

**Main findings:**

Diagnostic basis: Histologically, spindle cells, cytoplasmic vacuolation and vasculogenic characteristics. Immunochemically, strong positivity for CD31, CD34, and ERG supports an endothelial origin. The genetic biomarker WWTR1-CAMTA1 is present in most patients. The treatment strategy for patients is as follows: surgery is the preferred option for localized lesions (5-year survival rate > 80 %); antiangiogenic agents or chemotherapy can be used for those with multiorgan involvement, but the efficacy is limited. Prognostic risk factors included multiorgan metastasis, male sex, and age ≥ 55 years.

**Future directions:**

Precision treatment exploring targeted gene fusions (e.g., WWTR1-CAMTA1, YAP1-TFE3); research on the effects of Bartonella infection and EGFR overexpression on tumor progression; and the development of early diagnostic models based on radiomics.

## Highlights

• This is a rare low-to-moderate grade malignant tumor derived from vascular tissue.
• The tumor can present as bilateral multiple nodules or as a single cystic lesion.
• The histological features include spindle-shaped cells with vacuolated cytoplasm.
• The immunohistochemical markers CD31, CD34, and ERG are strongly positive.
• Surgical resection is performed for localized lesions, while anti-angiogenic therapy and chemotherapy are used for advanced metastatic lesions.

## Introduction

Pulmonary Epithelioid Haemangioendothelioma (PEH) is a low- to intermediate-grade malignant vascular mesenchymal tumour^1^ with an incidence of less than one in a million worldwide and accounts for < 1 % of all vascular tumours.^2^ Advancements in imaging technology and molecular pathology have improved the understanding of PEH, and the core challenges remain: Diagnostic difficulty: clinical manifestations (cough, chest pain) and features (multiple nodules in both lungs) lack specificity, leading to misdiagnosis rates as high as 60 %; Mechanistic obscurity: Tigenesis is associated with WWTR1-CAMTA1 gene fusion,^3^ but the core factors driving sex differences (80 % female) and wide age range (7–83 years) are unknown;^4^ and a lack of consensus in treatment: Current strategies rely on small retrospective studies (sample size < 50 cases), whereas surgical resection is the preferred option for localized disease (5-year survival rate > 80 %), and effective drugs for metastatic lesions are lacking.^5^ Currently, PEH is significantly fragmented: epidemiological data are limited to case reports, prognostic evaluation lacks a unified standard, and the impact of genetic heterogeneity on treatment has not been systematically explored. This review aims to summarize recent research progress on PEH and explore its clinical characteristics, diagnostic methods, and treatment strategies to enhance the understanding of PEH and help clinicians better manage and treat patients with PEH. By analysing the results of multiple studies, this study provides a reference basis for the clinical management of PEH and a path forward for future research directions.

### Clinical characteristics

#### Pathogenesis

The pathogenesis of PEH is still unclear and involves gene fusion and infection-related factors. Research has shown that the fusion of the WWTR1-CAMTA1 genes may promote tumorigenesis by regulating transcriptional activity, a phenomenon that has been reported in salivary gland epithelioid endothelial tumours.^6^ In addition, Antonescu et al.^7^ discovered a subgroup of YAP1-TFE3 gene fusions during the WWTR1-CAMTA1 gene fusion screening process for PEH. This subgroup exhibits unique morphological features, such as well-formed blood vessels, epithelioid cells in mature lumens, and abundant eosinophilic cytoplasm, and mainly appears in young patients.^7^^,^^8^

Chronic Bartonella infection is associated with the pathogenesis of PEH, and its underlying mechanism may involve the expression of vascular endothelial proliferation factor, which leads to abnormal proliferation of endothelial cells, cytoskeleton remodelling, and inhibition of apoptosis.^9^ Bartonella infection drives the malignant transformation of endothelial cells by upregulating proinflammatory genes and mitogenic signalling pathways (such as the vascular endothelial growth factor pathway). Bartonella is currently the only bacterial genus known to cause endothelial cell proliferation. These bacterial pathogens can upregulate mitotic and proinflammatory gene expression and lead to cytoskeleton reorganization and the inhibition of endothelial cell apoptosis. These findings suggest that these bacterial pathogens may contribute to the development of vascular tumours.^10^

Research has shown that abnormal activation of multiple signalling pathways in PEH cells is closely related to tumor proliferation, migration, and angiogenesis. Abnormal ErbB receptor signalling is also associated with tumor progression.^11^ Abnormal activation of the Vascular Endothelial Growth Factor (VEGF) signalling pathway is considered one of the main driving factors of angiogenesis in PEH.^12^ VEGF not only promotes the proliferation and migration of endothelial cells but also enhances vascular permeability in the tumor microenvironment, providing necessary nutrients and oxygen for tumor growth. In addition, studies have shown that the Wnt/β-catenin signalling pathway plays an important role in the occurrence of PEH. Abnormal activation of this pathway may lead to uncontrolled cell proliferation and promote the invasiveness of tumor cells.^13^ In PEH cells, upregulation of the Wnt signalling pathway is positively correlated with the malignancy of tumours, suggesting that this pathway may be a potential target for PEH therapy.

Moreover, the interaction between genes and the environment may affect the occurrence of PEH. For example, mutations in certain genes may increase an individual's sensitivity to environmental factors, thereby increasing the risk of tumours. One of the pathological features of PEH is the abnormal generation of blood vessels within the tumour, which is closely related to tumor growth and metastasis. Research has shown that PEH cells promote the generation of surrounding blood vessels by secreting various angiogenic factors, such as VEGF and FGF.^14^ These factors stimulate the proliferation and migration of endothelial cells, promote the formation of new blood vessels, and provide necessary nutrients and oxygen for tumor cells.

In addition, the Extracellular Matrix (ECM) components in the tumor microenvironment significantly affect the process of angiogenesis. Changes in the composition and structure of the ECM can affect the behaviour of endothelial cells and affect the efficiency of angiogenesis. For example, changes in the hardness and composition of the ECM may affect the migration and proliferation of endothelial cells through mechanical signals, thereby regulating angiogenesis.^15^

#### Clinical manifestations

In clinical practice, PEH is associated with a variety of symptoms, depending on the location and biological behaviour of the tumour. PEH in the lungs may present as asymptomatic or only mild respiratory symptoms, such as cough and chest pain in the early stages.^16^ As the condition progresses, a small number of patients may experience discomfort, such as alveolar haemorrhage, haemoptysis, anaemia,^17^ and occasionally clubbing fingers and weight loss. When the pleura is involved, it is often accompanied by pleural effusion.^18^ Nearly 50 % of patients have no symptoms, most of which are incidentally discovered during physical examinations.^19^ The clinical manifestations of this rare vascular tumor may vary depending on the location of the tumour. When PEH involves bone metastasis, there is a serious risk of pathological fracture. If this occurs in the vertebrae, it can lead to spinal compression, which results in sensory abnormalities, weakened muscle strength, and paraplegia.^20^

Imaging examinations typically reveal multiple small nodules with a diameter of less than 20 mm , and most patients can show bilateral lesions during imaging examinations.^21^ In addition, some patients may accidentally discover pulmonary nodules during physical examinations, indicating the potential occult nature of the disease.^22^ In one study, 71.4 % of patients presented with multiple small nodules, 22.9 % with multiple nodules and large lesions, and 5.7 % with a single lesion.^23^ The imaging features of PEH may also be accompanied by pleural thickening and calcification, especially in patients with large nodules.^24^ Owing to the overlap of these imaging features with those of other lung diseases, such as lung cancer and metastatic tumours, clinical doctors need to be cautious when interpreting imaging results and combine pathological examinations to confirm the diagnosis if necessary.

#### Physical signs and clinical staging

The signs of PEH usually vary depending on the location and size of the tumour. In the lungs, CT scans often reveal multiple small nodules, especially those distributed around blood vessels, that may be accompanied by pleural effusion or other lung lesions.^23^ In terms of clinical staging, PEH is usually divided into two types: limited and multiple. The former has a relatively better prognosis, whereas the latter may be associated with poorer survival rates. For example, in a previous study, patients with multiple lesions exhibited higher mortality rates during follow-up, particularly those with multiple lesions of the liver and lungs.^25^

#### Age and sex distributions of the patients

PEH patients have a wide age distribution, are usually between 20 and 80 years old, and are mainly middle-aged and elderly patients. Research shows that the proportion of female patients is greater than that of male patients, especially in cases involving the lungs and liver.^26^ For example, in a study of 7 cases of liver PEH, female patients accounted for the majority, with an average age of 45-years.^25^ In addition, sex differences may play an important role in the presentation and prognosis of diseases, and male patients may exhibit more severe symptoms and poorer prognoses in certain situations.^27^ Notably, although the liver and bones are the most commonly affected sites of PEH, the prognosis of pulmonary involvement is the worst,^28^ and elderly patients are more prone to multiple organ involvement. Notably, the superimposed effect composed of male sex and age ≥55 years increased the mortality rate by 3.1 times, suggesting that elderly men may need more active monitoring. This evidence collectively indicates that although PEH can occur at all ages, advanced age (especially ≥55 years) is an independent adverse prognostic factor, which may be related to changes in tumor biological behaviour or an increase in comorbidities.^3^ These data suggest that clinical doctors need to consider patients' sex and age when evaluating PEH to develop more personalized treatment plans.

### Pathological detection and diagnostic criteria

The diagnosis of PEH relies mainly on pathological examination, especially tissue biopsy. Pathological features include the epithelioid characteristics of tumor cells, which typically manifest as multinucleated cells with abundant cytoplasm.^29^ Macroscopically, the nodules range in diameter from several millimetres to 5 cm, with a cut surface that is greyish-white or yellowish-brown, hard in texture, and without a capsule. Microscopically, the nodules are clearly zoned, with abundant cells at the periphery and sparse cells in the centre, accompanied by coagulative necrosis, hyaline degeneration, amyloidosis, calcification, and even ossification. The tumor cells are round or polygonal, with an epithelioid morphology, are arranged in small nests, short cords, and glandular tubes, present as papillary or glomerular-like hyperplasia, and fill the alveolar cavities.^30^ The cytoplasm is abundant, eosinophilic or amphophilic,^31^ containing Weibel-Palade bodies; cytoplasmic vacuoles; and chondroid, mucoid, or hyaline degeneration of the matrix, which are characteristic structures of PEH and help to distinguish EHE from epithelioid angiosarcoma.^31^ Tumor tissue can invade small pulmonary arteries, pulmonary veins, and lymphatic vessels.

In immunohistochemical staining, PEH cells typically show positive reactions to CD31 and CD34, which confirms their vascular origin.^21^ The authors have included markers such as CD34 and D2–40 as diagnostic indicators, while markers like Ki-67 and p53 are now clearly identified as supportive in the diagnostic process. Currently, there are no unified diagnostic criteria for PEH, but according to the literature, a comprehensive assessment combining clinical manifestations, imaging features, and pathological results is an effective method for diagnosis.^22^ Some studies also suggest that when new pulmonary nodules are found by imaging examinations, PEH should be highly suspected and confirmed pathologically to avoid misdiagnosis.^22^ In summary, the diagnosis of PEH requires multidisciplinary cooperation and a comprehensive assessment combining clinical, imaging, and pathological aspects to improve the accuracy and timeliness of diagnosis.

### Imaging manifestations

#### X-Ray manifestations

With respect to pulmonary manifestations, the X-Ray features of PEH are usually blurry and may be similar to the imaging characteristics of other diseases. According to the literature, PEH often appears as multiple pulmonary nodules on X-Ray examination, and these nodules vary in size and shape, possibly appearing as circular or irregular shapes. Owing to the nonspecific nature of its imaging findings, the X-Ray diagnosis of PEH often requires differentiation from other diseases, such as primary or metastatic lymphadenocarcinoma, granulomatous infection, and diffuse interstitial lung disease.^16^ Therefore, when facing unexplained pulmonary nodules, clinical doctors need to consider the possibility of PEH and conduct a comprehensive evaluation on the basis of the patient's clinical manifestations.

#### CT scan features

CT plays a significant auxiliary role in the diagnosis of PEH. PEH typically manifests as multiple bilateral pulmonary nodules on CT scans, especially perivascular nodules, with a diameter usually less than 20 millimetres.^3^ CT images may also reveal the homogeneity and edge characteristics of the nodules. In some cases, extrapulmonary involvement of the liver or bones may be present. PET/CT examination revealed that PEH nodules usually exhibit increased FDG uptake, and there is a positive correlation between nodule size and the maximum standardized uptake value (SUVmax).^21^ These imaging characteristics provide important evidence for the early diagnosis and treatment planning of PEH.

#### Application of MRI in PEH

MRI is relatively less commonly used in the assessment of PEH, but it can provide valuable information in certain situations. The advantage of MRI lies in its high contrast for soft tissues, which enables it to more clearly display the boundaries of tumours and their relationships with surrounding tissues. Studies have shown that MRI can help evaluate the invasiveness of tumours and their relationship with surrounding structures, especially when the tumor is located in a complex anatomical position.^32^ Although MRI is not commonly used in the routine diagnosis of PEH, in specific cases, when combined with CT and X-Ray images, it can provide a more comprehensive assessment for clinical purposes and assist in the formulation of individualized treatment plans.

### Treatment strategy

Treatments for PEH vary and depend on the site and extent of tumor involvement, metastasis, and individual factors. Because PEH is rare, there is no universal treatment protocol.

#### Follow-up observation

For asymptomatic patients diagnosed with PEH by biopsy, regular follow-up observation every 3 to 6 months can achieve a better prognosis.^2^ Kitaichi et al.^33^ analysed 21 Asian patients with PEH, among whom 3 asymptomatic PEH patients did not receive any treatment, and the number and size of the nodules showed partial spontaneous remission during the follow-up period of 5–15 years.

#### Surgical treatment

Surgery plays a crucial role in cancer treatment, especially in the early stages of the disease. Surgery can not only remove the tumor directly but also obtain a tissue sample to conduct a pathological evaluation, which can help doctors formulate subsequent treatment plans. With the development of minimally invasive surgery technology, the indications and effects of surgery are expanding in the treatment of complex tumours. Minimally invasive surgery can reduce postoperative complications and improve patients' quality of life.^34^ If the lesion is small and the number of nodules is limited, surgery is the preferred treatment for PEH patients with one or more unilateral nodules, and complete surgical resection can achieve the best therapeutic effect. Moreover, Bagan et al.^17^ reported that pulmonary wedge resection has the same survival rate as anatomical lobectomy. However, owing to the small number of patients with lymph node metastasis, the prognostic value of hilar and mediastinal lymph node dissection remains unclear. Eguchi et al.^35^ reported a PEH patient with multiple pulmonary nodules in both lungs. Wedge resection of both lungs was performed on the patient, and a total of 32 pulmonary nodules were removed on both sides. The patient recovered well, and no tumor recurrence was observed during the 11-year follow-up.

#### Systemic treatment

Radiotherapy and chemotherapy are commonly used as adjuvant therapies in cancer treatment, and the selection of indications is usually based on the type and stage of the tumor and the specific situation of the patient. Radiation therapy can be used in many cases to control tumours locally, especially as an adjunct therapy after or before surgery. Studies have shown that postoperative radiotherapy can significantly improve survival rates for certain types of skin cancer, such as cutaneous squamous cell carcinoma and melanoma.^36^ Chemotherapy is mainly used for systemic treatment, especially if the tumor has metastasized. For some high-risk patients, such as patients with locally advanced non-small cell lung cancer, the combination of postoperative chemotherapy and radiotherapy is considered the standard treatment.^37^

For patients who cannot be cured surgically or who have extensive metastases, the choice of appropriate adjuvant therapy is critical. If the patient's bone is involved, radiotherapy can be chosen, and if the lesion involves deep soft tissue or internal organs, chemotherapy can be preferred.^38^ According to the literature, the chemotherapy drugs that can be used for PEH include carboplatin, paclitaxel, doxorubicin, vinorelbine, and gemcitabine. Carboplatin combined with paclitaxel is the most commonly used chemotherapy regimen at present.^39^ In recent years, given the general ineffectiveness of chemotherapeutic agents in PEH, antiangiogenic agents have become effective regimens for the treatment of metastatic PEH. In PEH patients, high expression of Vascular Endothelial Growth Factor (VEGF) is associated with tumor growth and metastasis^40^ Bevacizumab is a recombinant humanized monoclonal antibody that targets VEGF and has been successfully used in many types of malignant tumours.^40^ At present, bevacizumab combined with carboplatin and paclitaxel, as first-line treatments, can partially alleviate patients' conditions.^2^ Owing to the low incidence and sporadic distribution of PEH, the effectiveness and safety of chemotherapy regimens for the treatment of PEH still lack sufficient evidence-based medical support, and individual treatment regimens should be selected according to the specific conditions of patients.^41^ At present, with the increasing attention given to this disease, foreign countries have begun to focus on international multicentre clinical research on PEH, and I believe that there will be more and better drugs or treatment programs available to serve the clinic and patients.

#### Emerging therapies and clinical trials

In recent years, with the rapid development of biomedical technology, emerging therapeutic methods have gradually entered the stage of clinical application. These approaches, including immunotherapy, targeted therapy, and gene therapy, all show promising prospects. For example, immune checkpoint inhibitors have achieved remarkable efficacy in a variety of cancers (such as melanoma and non-small cell lung cancer) and can effectively improve patient survival.^41^ In addition, targeted drugs that target specific molecular targets, such as those for HER2-positive breast cancer, have also shown good efficacy and safety.^42^ The targeted therapy for PEH currently focuses on its angiogenic characteristics, with the inhibition of the Vascular Endothelial Growth Factor (VEGF) pathway at its core. The following are the key advancements and supporting evidence: Anti-angiogenic drugs: Bevacizumab: Mechanism of action: Targets VEGF-A, inhibits tumor angiogenesis.^43^ A retrospective study (*n* = 3) reported that 2 patients achieved stable disease for ≥ 6 months.^44^ Chemotherapy combined with targeted therapy: Bevacizumab + platinum-based chemotherapy: Case reports show that 67 % of patients achieved stable disease, with a median overall survival of 18 months.^45^ Clinical trials play an important role in the validation of these emerging treatments, helping doctors select the best treatment for their patients by comparing the effectiveness of new therapies with that of standard treatments. Although emerging treatments show promising therapeutic potential, more research and clinical trials are needed to evaluate their long-term effects and safety to provide patients with more personalized treatment options.

## Outlook

PEH is a rare malignant vascular tumor that is located between a completely benign haemangioma and a highly malignant haemangiosarcoma. Owing to its low incidence and lack of specificity in terms of clinical symptoms and imaging findings, PEH is often misdiagnosed or missed and does not receive appropriate treatment, resulting in a poor prognosis. Therefore, it is particularly important to clarify the pathogenesis and formulate standard treatment methods. The occurrence of this disease is associated with WWTR1-CAMTA1 gene fusion, YAP1-TFE3 gene fusion, and chronic Bartonella infection; thus, targeted therapy involving specific WWTR1-CAMTA1 gene fusion and YAP1-TFE3 gene fusion may have a considerable impact in the near future. If the presence of Bartonella infection is confirmed, eradicating the bacterial infection or blocking Bartonella's signalling for angiogenesis and cell proliferation may slow tumor progression and improve patient outcomes. In conclusion, PEH is relatively rare, and both the basic and clinical aspects of PEH are worthy of in-depth research. Improving the understanding of PEH is conducive to the correct diagnosis and early treatment of PEH.

## Ethics approval and consent to participate

The study is a review without ethical approval.

## Consent for publication

Not applicable.

## Availability of data and materials

The datasets used and analysed during the current study are available from the corresponding author upon reasonable request.

## Clinical trial number

Not applicable.

## Authors’ contributions

TB and ZSL analysed and interpreted the data regarding PEH. TB, XMZ, DJG, and LQX were responsible for part of the design of the paper. TB, WJS, ZSL and LQX performed for PEH. ZSL and LQX were major contributors to writing the manuscript. All authors read and approved the final manuscript.

## Funding

This work was supported by the National Natural Science Foundation of China for Young Scholars grants 82203317 (to Quanxing Liu); Hematopoietic Acute Radiation Syndrome Medical and Pharmaceutical Basic Research Innovation Center, Ministry of Education of the People’s Republic of China (RSBIC-B-202405); Chongqing Technology Innovation and Application Development Key Projects, Grant/Award Number: CSTB2022TIAD-CUX0019; and The Key Projects of Talent Incubation Plan of Xinqiao Hospital (2023YQB010); Chongqing Science and Health Joint Medical Science and Technology Innovation and Key Project (2025GGXM001); New Chongqing Youth Innovation Talent Project (Grant/Award Number: CSTB2024NSCQ-QCXMX0031).

## Conflicts of interest

The authors declare no conflicts of interest.
